# Supplementary material for: Association between oral microbiome and seven types of cancers in East Asian population: a two-sample Mendelian randomization analysis
Source: Front Mol Biosci. 2023 Nov 21;10:1327893. doi: 10.3389/fmolb.2023.1327893 (PMC10702768; doi:10.3389/fmolb.2023.1327893)
Supplement: Supplementary file 1 [file DataSheet1.ZIP › Supplementary Materials/MR plots of saliva/saliva═╝/Breast cancer/pheno.2558_to_breast cancer_forest.pdf]

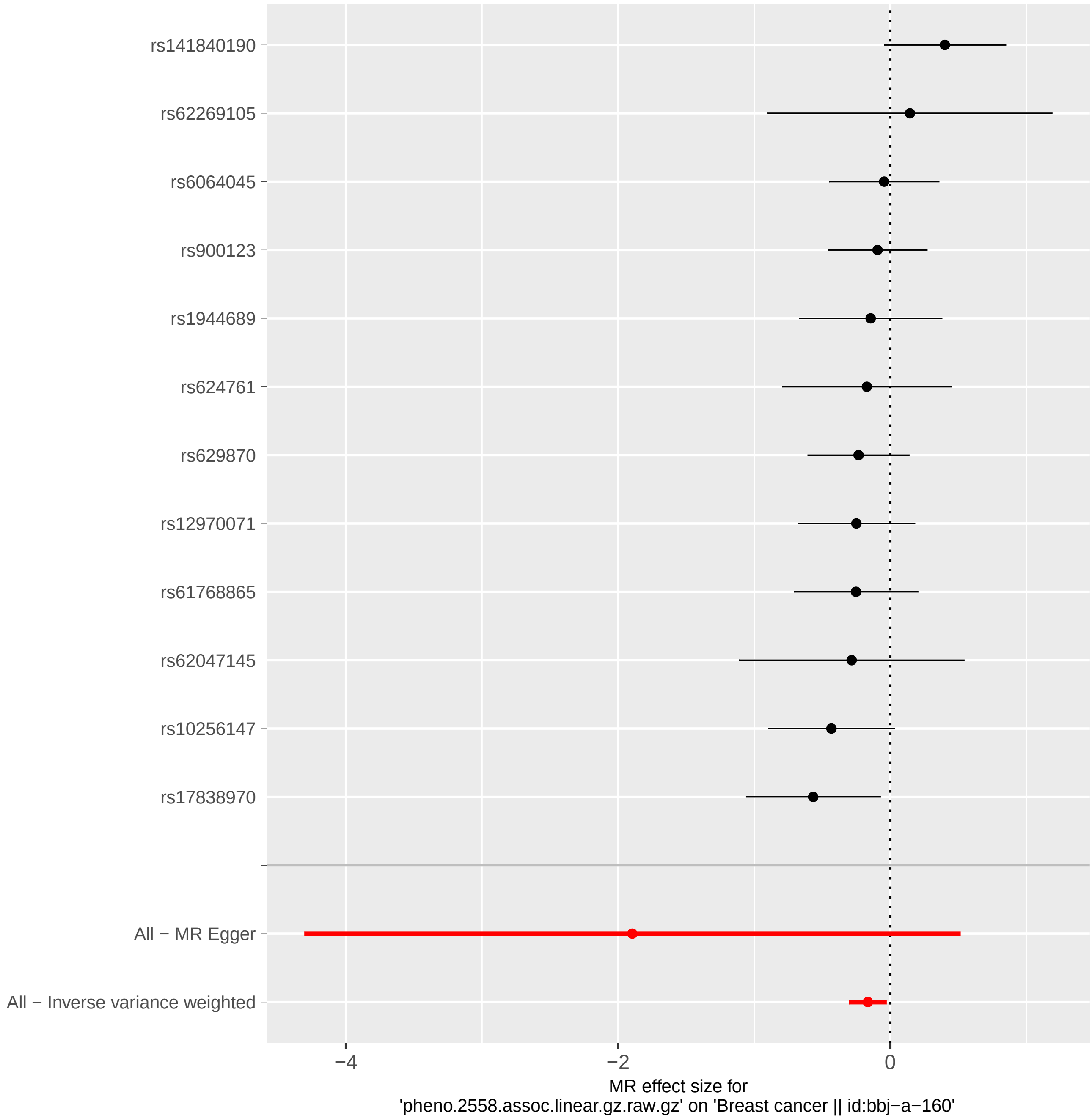
rs141840190
rs62269105
rs6064045
rs900123
rs1944689
rs624761
rs629870
rs12970071
rs61768865
rs62047145
rs10256147
rs17838970
All − MR Egger
All − Inverse variance weighted
−4
−2
0
MR effect size for
'pheno.2558.assoc.linear.gz.raw.gz' on 'Breast cancer || id:bbj−a−160'
